# Engineering of *Ogataea polymorpha* strains with ability for high-temperature alcoholic fermentation of cellobiose

**DOI:** 10.1093/femsyr/foae007

**Published:** 2024-02-23

**Authors:** Roksolana Vasylyshyn, Olena Dmytruk, Andriy Sybirnyy, Justyna Ruchała

**Affiliations:** Institute of Biotechnology, College of Natural Sciences, University of Rzeszow, Cwiklinskiej 2D Street, 35-601 Rzeszow, Poland; Department of Molecular Genetics and Biotechnology, Institute of Cell Biology NAN of Ukraine, Drahomanov Street 14/16, 79005 Lviv, Ukraine; Institute of Biotechnology, College of Natural Sciences, University of Rzeszow, Cwiklinskiej 2D Street, 35-601 Rzeszow, Poland; Department of Molecular Genetics and Biotechnology, Institute of Cell Biology NAN of Ukraine, Drahomanov Street 14/16, 79005 Lviv, Ukraine; Institute of Biotechnology, College of Natural Sciences, University of Rzeszow, Cwiklinskiej 2D Street, 35-601 Rzeszow, Poland; Department of Molecular Genetics and Biotechnology, Institute of Cell Biology NAN of Ukraine, Drahomanov Street 14/16, 79005 Lviv, Ukraine; Institute of Biotechnology, College of Natural Sciences, University of Rzeszow, Cwiklinskiej 2D Street, 35-601 Rzeszow, Poland; Department of Molecular Genetics and Biotechnology, Institute of Cell Biology NAN of Ukraine, Drahomanov Street 14/16, 79005 Lviv, Ukraine

**Keywords:** cellobiose, metabolic engineering, adaptive laboratory evolution

## Abstract

Successful conversion of cellulosic biomass into biofuels requires organisms capable of efficiently utilizing xylose as well as cellodextrins and glucose. *Ogataea (Hansenula) polymorpha* is the natural xylose-metabolizing organism and is one of the most thermotolerant yeasts known, with a maximum growth temperature above 50°C. Cellobiose-fermenting strains, derivatives of an improved ethanol producer from xylose *O. polymorpha* BEP/cat8∆, were constructed in this work by the introduction of heterologous genes encoding cellodextrin transporters (CDTs) and intracellular enzymes (β-glucosidase or cellobiose phosphorylase) that hydrolyze cellobiose. For this purpose, the genes *gh1-1* of β-glucosidase, *CDT-1m* and *CDT-2m* of cellodextrin transporters from *Neurospora crassa* and the *CBP* gene coding for cellobiose phosphorylase from *Saccharophagus degradans*, were successfully expressed in *O. polymorpha*. Through metabolic engineering and mutagenesis, strains BEP/cat8∆/gh1-1/CDT-1m and BEP/cat8∆/CBP-1/CDT-2mAM were developed, showing improved parameters for high-temperature alcoholic fermentation of cellobiose. The study highlights the need for further optimization to enhance ethanol yields and elucidate cellobiose metabolism intricacies in *O. polymorpha* yeast. This is the first report of the successful development of stable methylotrophic thermotolerant strains of *O. polymorpha* capable of coutilizing cellobiose, glucose, and xylose under high-temperature alcoholic fermentation conditions at 45°C.

## Teaser

Strains of xylose-fermenting yeast *Ogataea polymorpha* have been constructed capable of efficient cellobiose utilization and fermentation at elevated temperature (45°C).

## Introduction

Global ethanol production reached 120 billion liters per year, almost 80% of it was used as liquid fuel (https://www.ers.usda.gov/webdocs/outlooks/105762/bio-05.pdf?v=5239.1). However, because the main raw materials for the production of ethanol are starch and sucrose, which are also important components of food and feed for farm animals, this poses a significant ethical problem. Furthermore, bioethanol production from these raw materials may consequently lead to a reduction in their availability, an increase in their unit cost and, in some regions of the world, would lead to a shortage of these feedstocks (Bušić et al. 2018, Gong et al. 2022). As a result of the above, alternative sources are sought that could successfully replace sucrose and starch, while being inedible substrates; therefore, the main alternative is the dry plant biomass (lignocellulose) (Abo et al. 2019). The production of bioethanol from lignocellulose, a renewable and inexpensive raw material, is of great ecological and economic importance. However, existing technologies do not allow for cost-effective production as the efficiency of ethanol synthesis from several lignocellulose sugars is still too low. This problem mainly concerns pentoses such as xylose, l-arabinose, and the disaccharide, cellobiose (Robak and Balcerek 2020, Raj et al. 2022).

Cellobiose is the main product of the hydrolysis of cellulose by the cellulase enzyme complex (Lynd et al. 2002). Lignocellulose hydrolysates contain on average ~70% of cellodextrins (including cellobiose) and glucose and ~30% of xylose. Taking this into account, efficient conversion of lignocellulose to ethanol requires the use of organisms capable of efficient cellobiose fermentation (Bae et al. 2014, Oh et al. 2017). Most yeasts cannot ferment cellobiose because they lack cellobiose transporters and β-glucosidase capable of cellobiose hydrolysis to glucose (Bae et al. 2014, Oh et al. 2017).

Microorganisms metabolize cellobiose through three mechanisms:

1. Secretion of β-glucosidase and hydrolysis of cellobiose to glucose in the extracellular environment, followed by glucose transport to the cell (Singhania et al. 2013).
2. Production of cellodextrin transporters (CDTs) and cellobiose phosphorylases (CBPs). In this way cellobiose is phosphorylated into one molecule of glucose and one molecule of glucose-1-phosphate, which are subsequently metabolized in the glycolytic pathway. This mechanism is used by some species of the genus *Clostridium* (Demain et al. 2005).
3. Expression of CDTs and intracellular β-glucosidase to transport cellobiose into the cell, followed by hydrolysis of cellobiose to glucose molecules in the cytoplasm. This mechanism is used by fungi, e.g. *N. crassa* (Galazka et al. 2010).

Accordingly, cellulose hydrolysis by fungal cellulases first produces cellobiose, which can then be hydrolyzed to glucose by β-glucosidases. For these reasons, organisms that are able to effectively metabolize cellodextrins and glucose are sought to increase the efficiency of the production of bioethanol from plant biomass (Fan et al. 2016). Unfortunately, the high concentration of glucose in the environment inhibits the activity of cellulases. At the same time, one of the main drawbacks of the efficient conversion of sugars from the lignocellulosic hydrolysates could be an insufficient transport of cellobiose, which additionally is competitively inhibited by glucose. (Fan et al. 2016). Given this and the need to simultaneously utilize all lignocellulose sugars to ensure the cost-effectiveness of the process, direct fermentation of cellodextrins is a more appropriate approach.

The yeast *Saccharomyces cerevisiae* is a favored platform for microbial engineering efforts to produce biofuels from cellulosic hydrolysates because it is robust, simple to manipulate genetically, and it is capable of high carbon fluxes through central metabolic pathways (Zhang et al. 2011a). However, *S. cerevisiae* has a number of drawbacks, including an inability to naturally ferment pentose sugars (Hahn-Hagerdal et al. 2007), sensitivity to solvents (Ma and Liu 2010), and sensitivity to inhibitory compounds found in deconstructed plant materials (de Almeida et al. 2011). In this work, the yeast *Ogataea polymorpha* was used as a model organism. These methylotrophic, thermotolerant yeasts are among the best studied and, what is important, is that unlike conventional *S. cerevisiae*, it is naturally capable of fermenting xylose (Gellisen 2002, Ishchuk et al. 2009, Sibirny 2016, Ruchala and Sibirny 2021). We also proved that *O. polymorpha* is a promising organism for development as we constructed recombinant strains accumulating 40-fold elevated amounts of ethanol from xylose (Kurylenko et al. 2014, 2018, 2021, Ruchala et al. 2017, Vasylyshyn et al. 2020).

**Figure 1.**
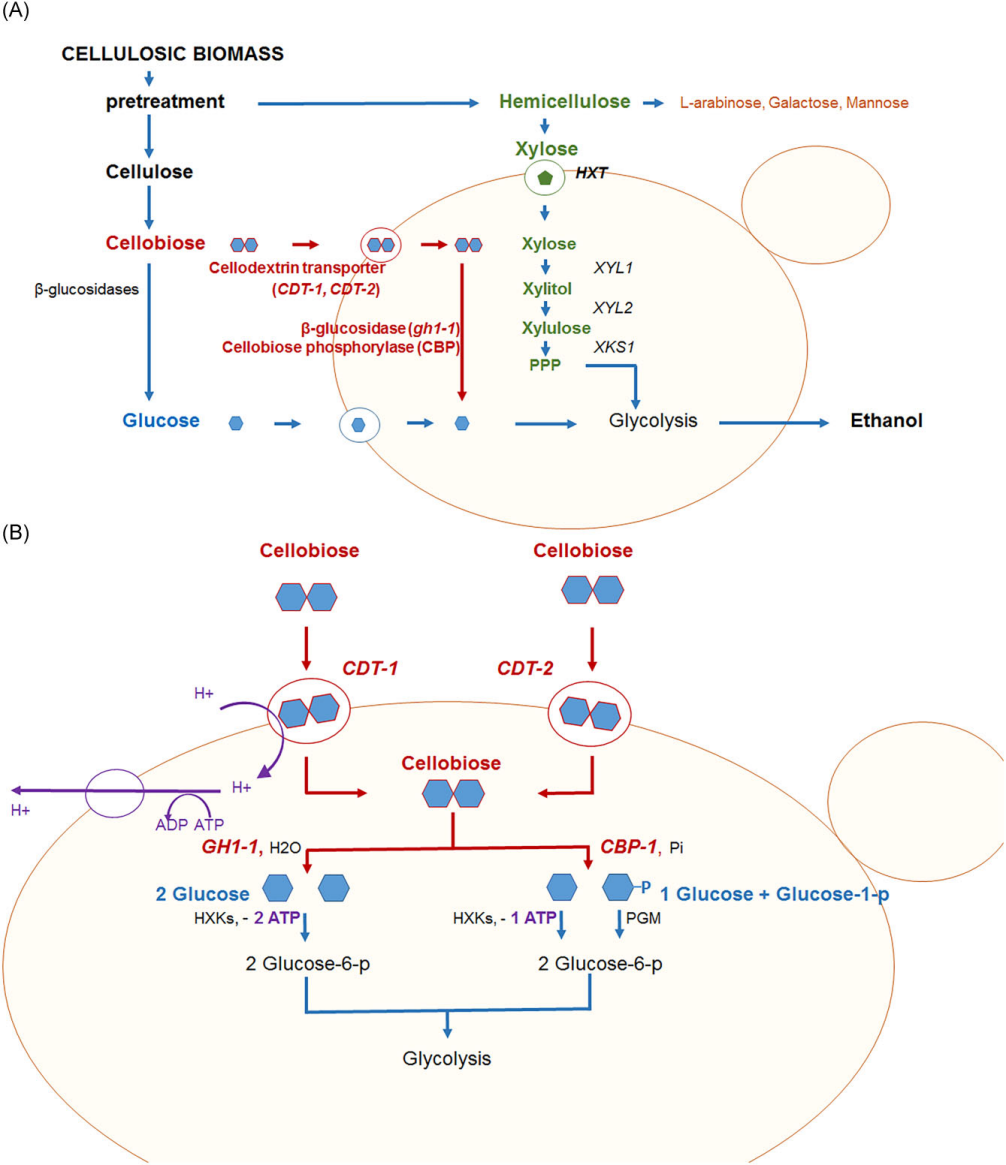
Scheme of simultaneous cofermentation of cellobiose and xylose without glucose repression. (A) A method of improving an *O. polymorpha* strain (BEP/*cat8Δ*) to create yeast capable of fermenting two sugars by heterologous expression. (B) Variants of cellobiose uptake pathways consisting of CDT (cdt-1 or cdt-2) and intracellular β-glucosidase (gh1-1) from the filamentous fungus *N. crassa* or CBP from *Saccharophagus degradans*.

However, one of the disadvantages in using *O. polymorpha* for producing cellulosic biofuels is its inability to naturally ferment cellodextrins such as cellobiose. Cellobiose, the repeating unit of cellulose, is a β(1–4) linked disaccharide of glucose, i.e. produced by the enzymatic digestion of cellulose by cellulases (Zhang and Lynd 2004). The main goal of the current study was the construction of *O. polymorpha* strains capable of cellobiose fermentation. There are two known cellobiases (enzymes that break down cellobiose into two glucose molecules). The first is gh1-1 regular cellobiase (called also β-glucosidase), which produces two glucose molecules (Znameroski et al. 2012). The second cellobiase, (known as CBP or cellobiose phosphorylase), is an intracellular enzyme generally found in anaerobic bacteria that cleaves the cellobiose to glucose and glucose-1-phosphate, providing energetic advantages under the anaerobic conditions required for large-scale biofuel production (Fig. 1) (Zhang et al. 2011b). To successfully convert cellobiose, it is also necessary to ensure efficient transport of this sugar into the cell. Cellobiose is transported across the membrane by either CDT-1 (active transporter consuming one ATP per cellobiose) or CDT-2 (energy-independent facilitator) (Kim et al. 2014, Madej et al. 2014, Kell et al. 2015). Previously, the kinetic properties of the respective transporters were improved through laboratory evolution in *S. cerevisiae* yeast. As a result of the F213 L mutation in CDT-1 and the N306I mutation in CDT-2, the overall expression, stability, and cellobiose transport were enhanced. For example, *S. cerevisiae* yeast expressing N309I CDT-2 showed ~6-fold greater intracellular accumulation of cellobiose than engineered yeast expressing CDT-2 (Lee and Jin 2017, Choi et al. 2022a).

This study aimed to evaluate whether expression of codon optimized genes from *N. crassa gh1-1* (encoding β-glucosidase), *CDT-1m* [encoding mutant CDT (F213L)], *CDT-2m* [encoding mutant CDT (N306I)], and from *Saccharophagus degradans CBP* (encoding CBP) will make *O. polymorpha* capable of utilizing and fermenting cellobiose (Fig. 1).

## Material and methods

### Strains, media, and culture conditions

*Ogataea polymorpha* BEP/cat8Δ strain (Ruchala et al. 2017) was grown on YPD (10 g/l yeast extract, 10 g/l peptone, and 20 g/l glucose) or minimal medium (6.7 g/l YNB without amino acids, 20 g/l glucose) at 37°C. For selection of yeast transformants on YPD, 0.1 g/l of nourseothricin were added.

The *Escherichia coli* DH5α strain [Φ80d*lacZ*ΔM15, *recA*1, *endA*1, *gyrA*96, *thi*-1, *hsdR*17(r^−^_K_, m^+^_K_), *supE*44, *relA*1, *deoR*, Δ(*lacZYA*–*argF*)U169] was used as a host for plasmid propagation. Strain DH5α was grown at 37°C in LB medium as described previously (Sambrook et al. 2001). Transformed *E. coli* cells were maintained on a medium containing 100 mg/l of ampicillin.

### Construction of *O. polymorpha* strains with overexpression of the *gh1-1, CBP, CDT-1m*, and *CDT-2m* genes

The following genes were selected and optimized for work: *gh1-1* gene from *N. crassa* encoding β-glucosidase; *CBP* gene from *S. degradans* encoding CBP; *CDT-1m* gene from *N. crassa* encoding mutant CDT (F213L); *CDT-2m* gene from *N. crassa* encoding original CDT (N306I) (Kim et al. 2018).

**Figure 2.**
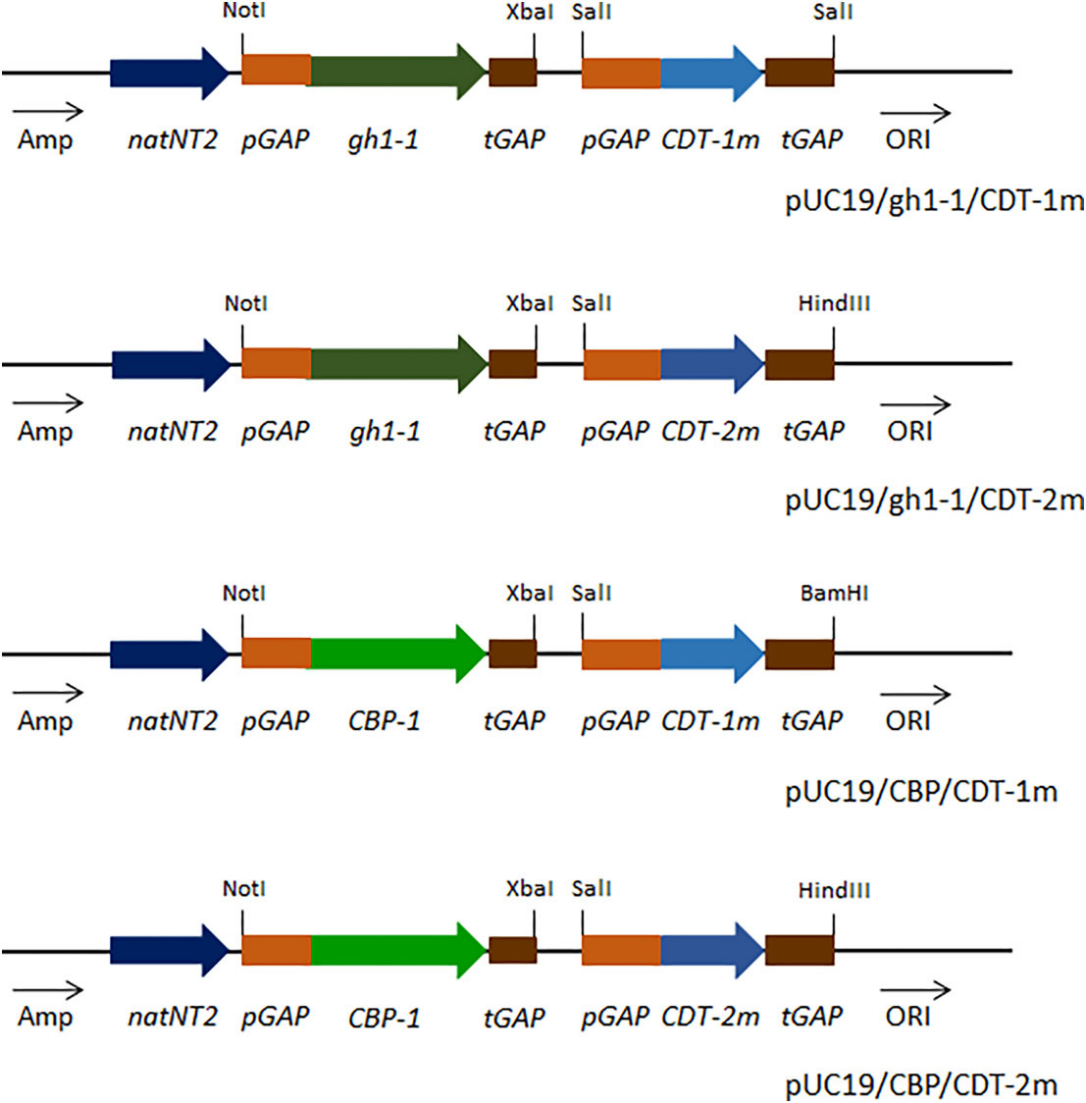
Schemes of plasmids for simultaneous overexpression of *gh1-1, CBP-1, CDT-1m*, and *CDT-2m* genes under control of *GAP1* promoter.

Optimization of genes sequences was performed according to codon usage of *O. polymorpha* using online resource http://atgme.org/?i=3. (Table S1, Supporting Information). The *gh1-1, CBP, CDT-1m*, and *CDT-2m* genes were overexpressed under control of strong constitutive *GAP1* promoter of glyceraldehyde-3-phosphate dehydrogenase gene in the frame of the single copy plasmid pUC19/pGAP/NTC (Vasylyshyn et al. 2020). For this, ORF of genes gh1-1, CBP, CDT-1m, and CDT-2m were flanked with sites for restriction enzymes XbaI and NotI. Expression modules for genes *gh1-1* and *CDT-1m* or *CDT-2m* as well as *CBP* and *CDT-1m* or *CDT-2m* were combined on single plasmid, using primers Ko1330/Ko1332 for amplification pGAP_CDT1_tGAP from plasmid pUC19_NTC_pGAP_CDT1_tGAP and cloned into SalI site of the plasmid pUC19_pGAP_gh1-1_tGAP_NTC, Ko1328/Ko1329 for amplification pGAP_CDT-1_tGAP from plasmid pUC19_NTC_pGAP_CDT-1_tGAP. PCR-product was SacI/BamHI-degested and cloned into SacI/BamHI-degested plasmid pUC19_pGAP_CBP_tGAP_NTC, Ko1130/Ko1131 for amplification pGAP_CDT-2_tGAP from plasmid pUC19_NTC_pGAP_CDT-2_tGAP. PCR-product was SalI/HindIII-degested and cloned into SalI/HindIII-degested plasmid pUC19_pGAP_gh1-1_tGAP_NTC and for amplification pGAP_CDT-2_tGAP from plasmid pUC19_NTC_pGAP_CDT-2_tGAP. PCR-product was SalI/HindIII-degested and cloned into SalI/HindIII-degested plasmid pUC19_pGAP_CBP-1_tGAP_NTC. The final constructed plasmids were named pUC19/gh1-1/CDT-1m (Fig. 2A), pUC19/gh1-1/CDT-2m (Fig. 2B), pUC19/CBP/CDT-1m (Fig. 2C), and pUC19/CBP/CDT-2m (Fig. 2D). These plasmids were introduced into the genome of *O. polymorpha* BEP*/*cat8∆ by electroporation. Transformants were selected on solid YPD medium containing nourseothricin with a final concentration of 0.1 g/l. Selected transformants were stabilized by alternating cultivation in nonselective and selective media and examined by diagnostic PCR using pairs of primers, respectively, Ko1224/Ko1237, Ko 1224/Ko1238, Ko 1224/1239, Ko1224/Ko1240 (Table S2 and Figure S1, Supporting Information). The resulting stable recombinant strains were grown at 37°C in liquid YNB medium with 2% cellobiose instead of glucose, followed by determining the rate of biomass accumulation and in liquid YNB medium with 10% cellobiose during 120 h at 45°C followed by establishing the level of ethanol production. All strains were characterized by a similar growth rate and level of ethanol accumulated. The best-performing transformants were used for subsequent analyses.

### Adaptive laboratory evolution as an approach for phenotype improvement

To enhance BEP/cat8∆/gh1-1/CDT-2m, BEP/cat8∆/CBP-1/CDT-2m productivity in cellobiose transport, cellobiose nonfermenting strains underwent adaptive evolution through sequential periodic fermentations under conditions where cellobiose served as not only the main carbon source, but also as a selective pressure. Initially, cell suspensions were added to YNB minimal medium containing 10% cellobiose so that the final cell concentration in the medium after inoculation was 0.1 OD_590_. After 10 days of cultivation, the number of cells reached 2.3 mg/ml. The cells were transferred to fresh YNB medium with 10% cellobiose and incubated for the next 10 days at 37°C. The starting OD_590_ at the beginning of the next round was similarly likewise 0.1 OD_590_. In addition, a sample of cells after each of the six rounds (cells were harvested on the 10th, 20th, 30th, 40th, 50th, and 60th day) was taken for further analysis of the rate of biomass accumulation and the level of ethanol production under the conditions of high-temperature alcoholic fermentation. Subsequently, from the last culture showing the best growth dynamics, individual colonies were isolated, and their fermentation ability compared to the parental strains to confirm improvement. All isolated colonies from the adaptive culture demonstrated enhanced fermentation of 10% cellobiose at 45°C compared to the parental strains. Consequently, cells with beneficial cellobiose metabolism mutations would become dominant during serial culturing.

### Random selection of the mutants with improved cellobiose alcoholic fermentation through ultraviolet light and chemical mutagenesis

To initiate a mutagenesis, a fresh subculture of cells grown into log phase is collected, washed, and resuspended in potassium phosphate buffer (Barbour et al. 2006). Irradiation was carried out with 1 ml of cell suspension (inoculum OD_590_ 0.1–0.3), which was added to a plate (ø 67 mm) and placed under an ultraviolet (UV) lamp [UltraViol NBV15N ∼230 V, 50 Hz (typ B), 25VA (IP20)] at a height of 10 cm. Irradiation lasted 35 s with constant stirring of the suspension. Afterward, the irradiated cells were kept in the dark for 40 min to avoid photo reactivation, and then plated onto selective YNB medium containing 0.5% cellobiose for initial screening of the phenotype, and onto YNB medium with 1% cellobiose and 200 mg/l 2-Deoxy-D-glucose (2-DG) for better phenotype tracking. The addition of 2-DG reduces the availability of energy from glucose for the yeast. The application of 2-DG in experiments can prompt yeast to rely more on other energy sources, such as cellobiose. As a result, cells may alter their metabolic pathways, including the activation of cellobiose phosphorolysis routes. A total of 30 colonies with the largest size were chosen and cultivated in YPD medium for 36 h, and then each colony was inoculated into 3 ml YNB medium with 10% cellobiose in rubber-sealed test tubes at an initial OD_590_ of 0.1. All the test tubes were cultivated for 72 h (45°C and 140 rpm). The metabolites were analyzed regularly after cultivation and the colony with the highest ethanol production rate and cellobiose consumption rate was isolated.

### Batch fermentation

Yeast cells were cultivated in YP with 2% cellobiose medium to prepare inoculums for fermentation and cells were harvested by centrifugation (4000 rpm, 5 min) at mid-exponential phase. Flask fermentation experiments were performed in 40 ml YNB medium containing 10% cellobiose; 8% cellobiose and 4% xylose; 5% cellobiose, 4% xylose, and 5% glucose (Carroll and Somerville 2009, Pendse et al. 2023) using 100 ml shaking flask under oxygen limited conditions (45°C, 140 rpm). The initial biomass concentration for fermentation experiments was 2 g (dry weight)/l. Fermentations were repeated at least in three independent experiments.

### Analyses

The biomass was determined turbidimetrically (dry weight) with a Helios Gamma spectrophotometer (OD, 590 nm; cuvette, 10 mm) with gravimetric calibration. Concentrations of xylose and ethanol from fermentation in medium broth were analyzed by HPLC (PerkinElmer, Series 2000, USA) with an Aminex HPX-87H ion-exchange column (Bio-Rad, Hercules, USA). A mobile phase of 4 mM H_2_SO_4_ was used at a flow rate 0.6 ml/min and the column temperature was 30°C. Experiments were performed at least twice.

## Results

### Adaptive laboratory evolution and selection of *O. polymorpha* strains with heterologous expression of the *gh1-1, CBP* genes, and modified version of the CDT-2m transporter

**Figure 3.**
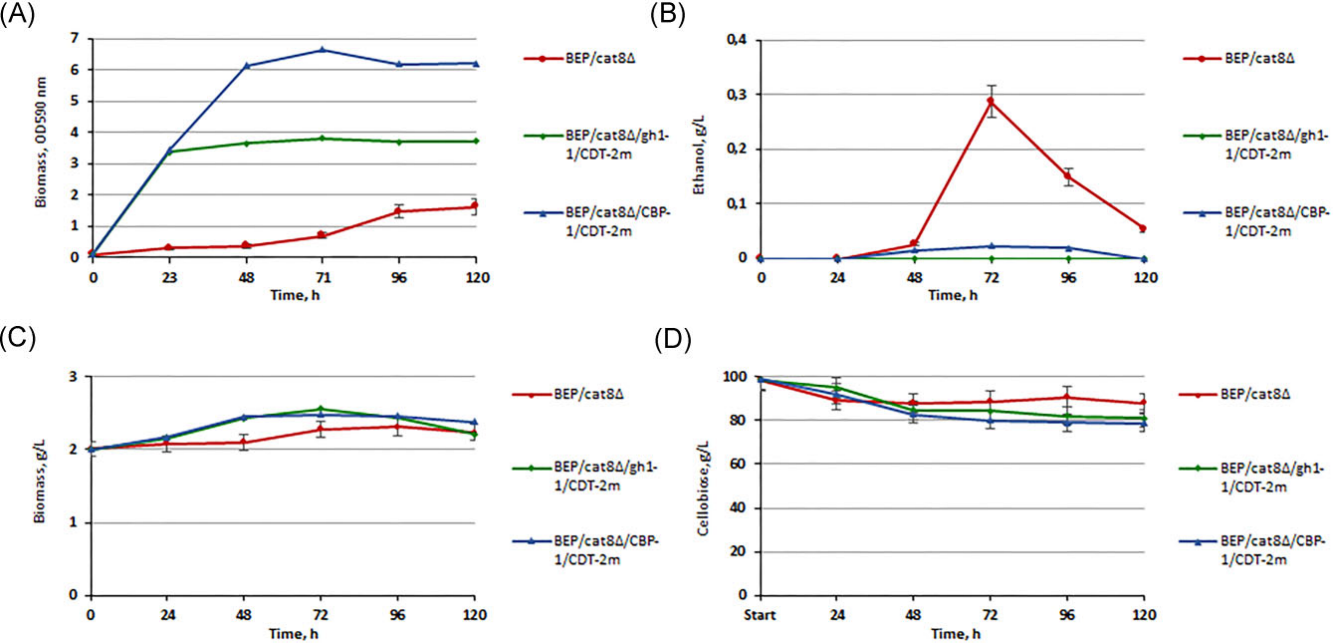
Biomass accumulation (A) and ethanol production (B) of *O. polymorpha* BEP/cat8∆ and recombinant strains with *gh1-1/CDT-2m, CBP-1/CDT-2m* simultaneous overexpression genes during growth test on 2% сellobiose at 37°C and alcoholic fermentation 10% сellobiose at 45°C. Biomass accumulation (C) and sugar consumption (D) of *O. polymorpha* BEP/cat8∆ and recombinant strains with *gh1-1/CDT-1m, CBP-1/CDT-1m* simultaneous overexpression genes during the alcoholic fermentation of 10% cellobiose at 45°C. Data are shown as mean of four independent experiments.

We selected the thermotolerant methylotrophic yeast *O. polymorpha* as a model organism to study the metabolism and fermentation of cellobiose due to the numerous advantages described above, however, one of the disadvantages is its inability to naturally ferment cellodextrins such as cellobiose. In our previous study, the advanced *O. polymorpha* (BEP/cat8Δ) ethanol producer from xylose was isolated by a combination of methods of metabolic engineering and classical selection (Ruchala et al. 2017). The BEP/cat8Δ was used as a recipient to evaluate and compare the impact of the introduction of the overexpressed heterologous ß-glucosidase, CBP and CDT-2m transporters on sugar consumption and alcoholic fermentation performance. To achieve this goal, vectors to overexpress pUC19/gh1-1/CDT-2m and pUC19/CBP/CDT-2m have been introduced into genome of BEP/*cat8Δ* under control of the strong constitutive *GAP* promoter. The modified versions of heterologous transporters from *N. crassa* were obtained thanks to the complex synthesis of genes by the biotechnology company GenScript Biotech. The CDT-2m had a substitution of asparagine to isoleucine at position 306. This mutation in the yeast *S. cerevisiae* has been described to increase the cellobiose uptake rate and the stability of CDT-2. (Lee and Jin 2017, Kim et al. 2018), (Table S1, Supporting Information). The impact of modifications on growth dynamics in a medium with 2% cellobiose and the level of ethanol production during high-temperature yeast fermentation of 10% cellobiose (45°C) were analyzed in the obtained recombinant strains with overexpression of the gene pairs gh1-1/CDT-2m and CBP-1/CDT-2m. The study of the growth (Fig. 3A) was carried out on YNB minimal liquid medium with 2% cellobiose (initial OD_590_ of 0,1). BEP/cat8∆/CBP-1/CDT-2m transformants, whose metabolic pathway requires only one molecule of ATP, and demonstrated significantly higher levels of biomass accumulation, compared to BEP/cat8∆/gh1-1/CDT-2m strains overexpressing ß-glucosidase, which use two moles of ATP. Further analysis of recombinant strains consisted of determining the efficiency of alcoholic fermentation. It was established that the combinations of the corresponding genes, despite the fact that the sugar consumption rate in the respective transformants was higher than in the parental strain (Fig. 3D), did not result in the production of ethanol from cellobiose, which was an unexpected result for us. (Fig. 3B and C).

**Figure 4.**
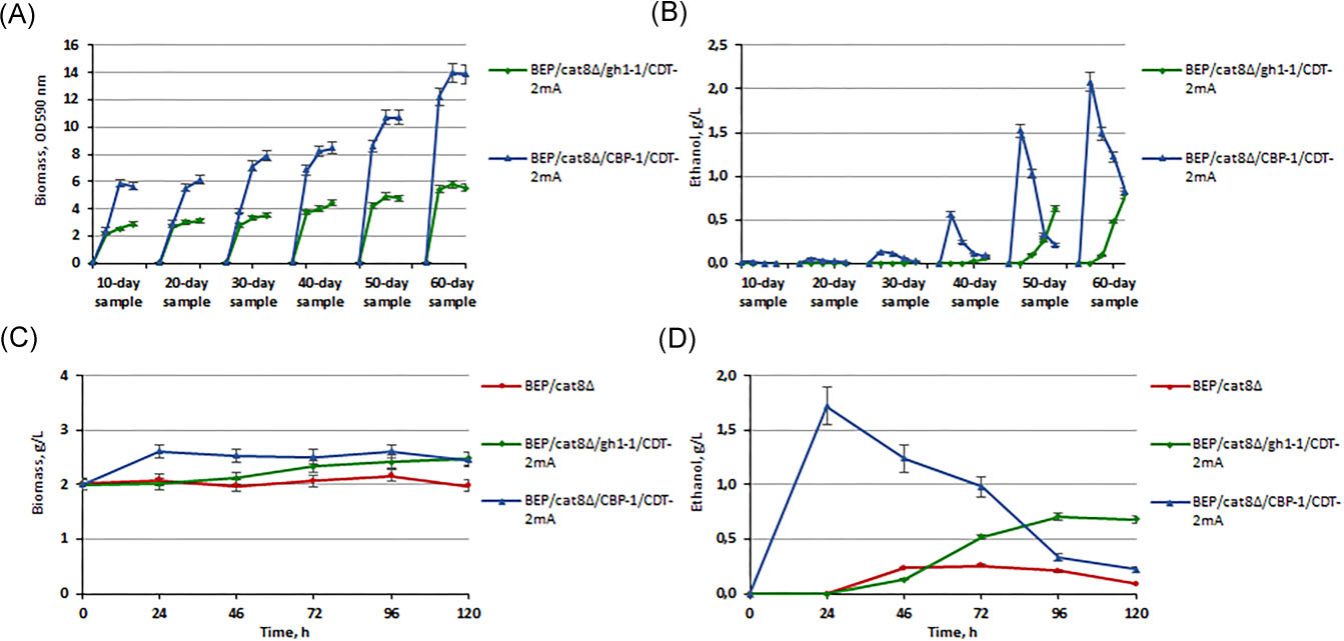
Biomass accumulation on 2% сellobiose at 37°C (A) and ethanol production from 10% сellobiose at 45°C (B) of *O. polymorpha* recombinant strains with *gh1-1/CDT-2m, CBP-1/CDT-2m* simultaneous overexpression genes during adaptive laboratory evolution. Biomass accumulation (C) and ethanol production (D) of *O. polymorpha* BEP/cat8∆/gh1-1/CDT-2mA, BEP/cat8∆/CBP-1/CDT-2mA strains during the alcoholic fermentation of 10% cellobiose at 45°C after adaptive laboratory evolution. «A»—mutants obtained by laboratory evolution.

**Figure 5.**
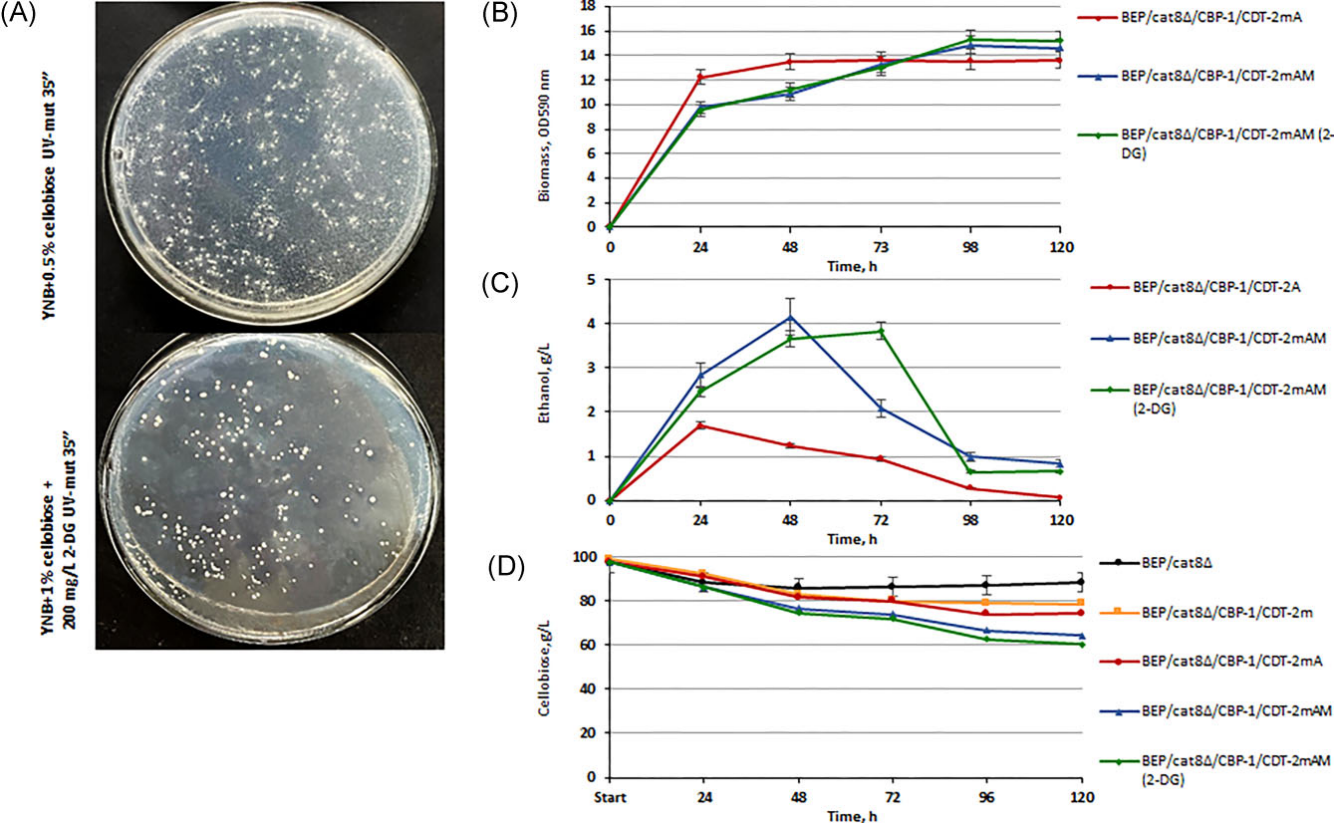
Selection of *O. polymorpha* BEP/cat8∆/CBP-1/CDT-2mA mutants after UV mutagenesis on medium with 0,5% cellobiose or 2-DG and 1% cellobiose as the only carbon source (A). Biomass accumulation during growth test on 2% сellobiose at 37°C (B), ethanol production (C), and sugar consumption (D) of *O. polymorpha* BEP/cat8∆/CBP-1/CDT-2mAM strains during the alcoholic fermentation of 10% cellobiose at 45°C. «A»—mutants obtained by laboratory evolution, «AM»—mutants obtained in two stages as a result of laboratory evolution, and UV—mutagenesis with 2-DG as a glycolysis inhibitor.

One of the effective (although poorly researched) opportunities to activate the alcoholic fermentation process is the use of adaptive laboratory evolution (Mans et al. 2018). Therefore, this study was conducted to enhance the transport ability of CDT-2m as a result of the direct or indirect effect of accumulated genomic adaptive changes. The experiments were based on long-term serial batch transfer fermentation (Sauer 2001, Chen et al. 2023). We imposed cellobiose as a selection pressure onto yeast expressing CDT-2m and the CBP or CDT-2m and the β-glucosidase. After six rounds of serial subcultures on cellobiose, we isolated an evolved strain exhibiting significantly faster accumulating biomass on cellobiose (Fig. 4A and C) and increased ethanol yields (Fig. 4B and D). It is worth noting that BEP/cat8∆/CBP-1/CDT-2mA strains (A—mutants obtained by laboratory evolution) underwent changes much faster, and their ethanol production level during high-temperature alcoholic fermentation (45°C) of 10% cellobiose reached 1.7 g/l. In contrast, BEP/cat8∆/gh1-1/CDT-2mA strains achieved only 0.6 g ethanol/l after 2 months of adaptation. Furthermore, we report here that following experiments involving UV mutagenesis and 2-deoxyglucose treatment (Fig. 5A) of the BEP/cat8∆/CBP-1/CDT-2mA strain also allowed the generation of mutants BEP/cat8∆/CBP-1/CDT-2mAM (AM—mutants obtained in two stages as a result of laboratory evolution and UV—mutagenesis with 2-DG as a glycolysis inhibitor). These mutants are characterized by improved biomass accumulation dynamics, sugar consumption rates and higher ethanol production levels from cellobiose to 4.2 g/l (Fig. 5B–D). Consequently, with previous successes of improving transporter properties by laboratory evolution (Ha et al. 2013b, Lian et al. 2014), a similar strategy was adapted to improving BEP/cat8∆/CBP-1/CDT-2mAM strain. We hypothesized that the prior introduction of heterologous genes involved in cellobiose metabolism into *O. polymorpha* yeast significantly alleviated the selective pressure of cellobiose. Laboratory evolution and mutagenesis meanwhile allowed for the reprogramming of intracellular metabolic processes to achieve the activation of cellobiose alcoholic fermentation in the respective strains.

### Heterologous expression of the *gh1-1, CBP* genes, and a modified version of the CDT-1m, transporter in *O. polymorpha*

**Figure 6.**
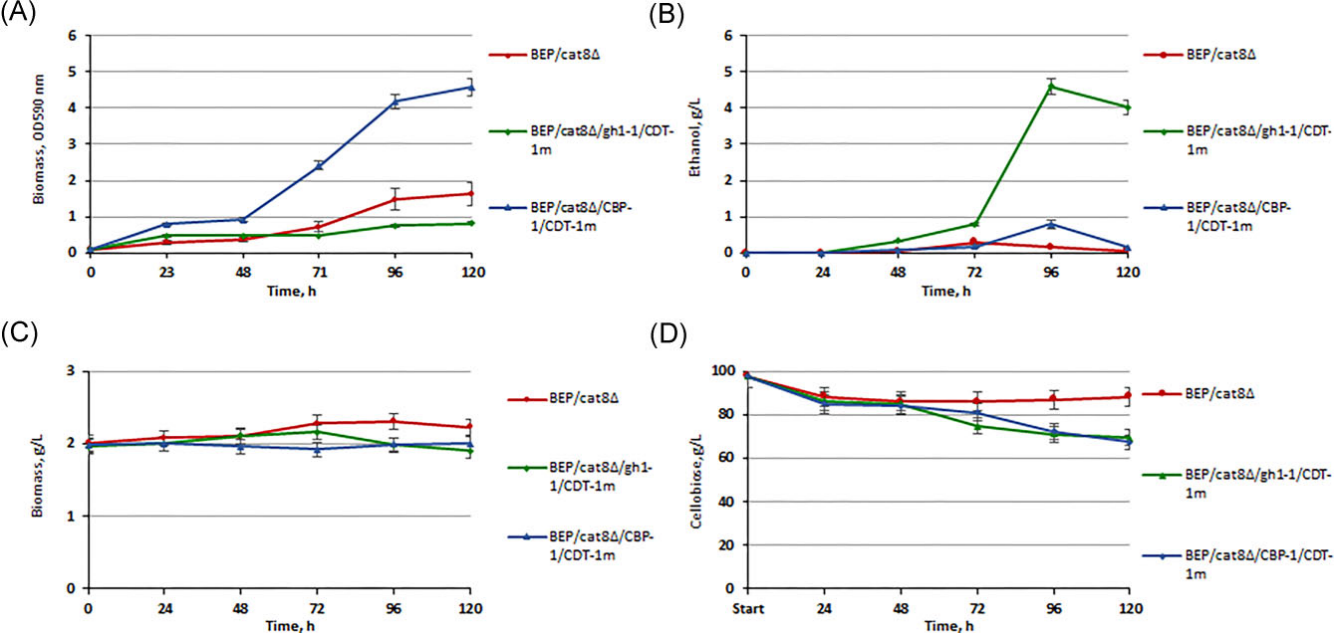
Biomass accumulation (A) and ethanol production (B) of *O. polymorpha* BEP/cat8∆ and recombinant strains with *gh1-1/CDT-1m, CBP-1/CDT-1m* simultaneous overexpression genes during growth test on 2% сellobiose at 37°C and alcoholic fermentation 10% сellobiose at 45°C. Biomass accumulation (C) and sugar consumption (D) of *O. polymorpha* BEP/cat8∆ and recombinant strains with *gh1-1/CDT-1m, CBP-1/CDT-1m* simultaneous overexpression genes during the alcoholic fermentation of 10% cellobiose at 45°C. Data are shown as mean of four independent experiments.

Previously, it was reported that *S. cerevisiae* yeast expressing CDT-2m could not efficiently utilize cellobiose compared to yeast expressing CDT-1m (Kim et al. 2014). For this reason, vectors were constructed for the overexpression of β-glucosidase or CBP in complex with the heterologous modified transporter CDT-1m. The CDT-1m was modified by the substitution of phenylalanine to leucine at position 213. This single mutation in the transporter accounts for the faster and more efficient fermentation of cellobiose in recombinant strains of *S. cerevisiae* (Lee and Jin 2017), (Table S1, Supporting Information). The impact of changes on growth dynamics in a medium with 2% cellobiose and the level of ethanol production during 10% cellobiose high-temperature yeast fermentation (45°C) in the obtained recombinant strains with overexpression of *gh1-1/CDT-1m* and *CBP-1/CDT-1m* was analyzed. The study of the growth (Fig. 6A) of the obtained strains with overexpression gene pairs in comparison with the corresponding parental strains was carried out on YNB minimal liquid medium with 2% cellobiose (OD_590_ 0.1).

The most energy-consuming pathway (3 moles of ATP) involves the simultaneous overexpression of β-glucosidase and the CDT-1m symporter. Under the growth test conditions (220 rpm, 37°C), the BEP/cat8∆/gh1-1/CDT-1m transformants exhibited minor biomass accumulation in the cellobiose-containing medium. Furthermore, such transformants showed impaired growth on glucose and other tested sugars (results not shown). It is also important to mention that BEP/cat8∆/CBP-1/CDT-1m transformants with CBP and transporter CDT-1m overexpression exhibited a significantly prolonged lag-phase and slower biomass accumulation rates, reaching the stationary phase at 120 h of growth test, whereas BEP/cat8∆/CBP-1/CDT-2m strains, even without adaptive changes, with CBP and transporter CDT-2m reached a stationary phase at about 24 h. Analyzing the effect of these approaches, we concluded that replacing the heterologous hydrolytic pathway of cellobiose utilization with a heterologous phosphorolytic pathway in *O. polymorpha* resulted in higher biomass yields, probably due to increased free energy (ATP) conservation. It was hypothesized that this could also have a significant impact during alcoholic fermentation under oxygen limitation conditions, because the difference between the hydrolytic and phosphorolytic pathways is important for cellular energetics. However, it has been established that the BEP/cat8∆/gh1-1/CDT-1m transformants (Fig. 6B and C) are characterized by the highest level of ethanol production among the obtained variants, reaching 5 g of ethanol/l at 96 h of high-temperature alcohol fermentation (45°C) with 10% cellobiose, while the BEP/cat8∆/CBP-1/CDT-1m strains produced a minor amount of ethanol, ~1 g/l. Interestingly, the sugar level in the medium at the end of fermentation (120 h) was ~70% in both strains (Fig. 6D). Referring to the literature, the slow and inefficient cellobiose fermentation can possibly be explained by a change in the activity of glucose-phosphorylating enzymes observed during the utilization of nonfermentable carbon sources, leading to the potential accumulation of glycolysis products, such as glucose-6-phosphate and fructose-6-phosphate. (Lin et al. 2014, Chomvong et al. 2017a).

These results suggest that CDT-1m is a more efficient transporter of cellobiose than CDT-2m for *O. polymorpha* in facilitating cellobiose fermentation. Overexpression of CDT-1m promoted high-temperature alcoholic fermentation of cellobiose without additional adaptive approaches, but still with low efficiency. Obtaining the corresponding result, we adhere to the belief that one of the approaches to enhance cellobiose transport efficiency is the expression of heterologous transporters with high affinity to this sugar in *O. polymorpha*. However, the problem of this approach is ensuring the correct localization of heterologous proteins in the cytoplasmic membrane (Vasylyshyn et al. 2020).

### Consumption and alcoholic fermentation of a sugar mixture by obtained recombinant and mutant *O. polymorpha* strains

**Figure 7.**
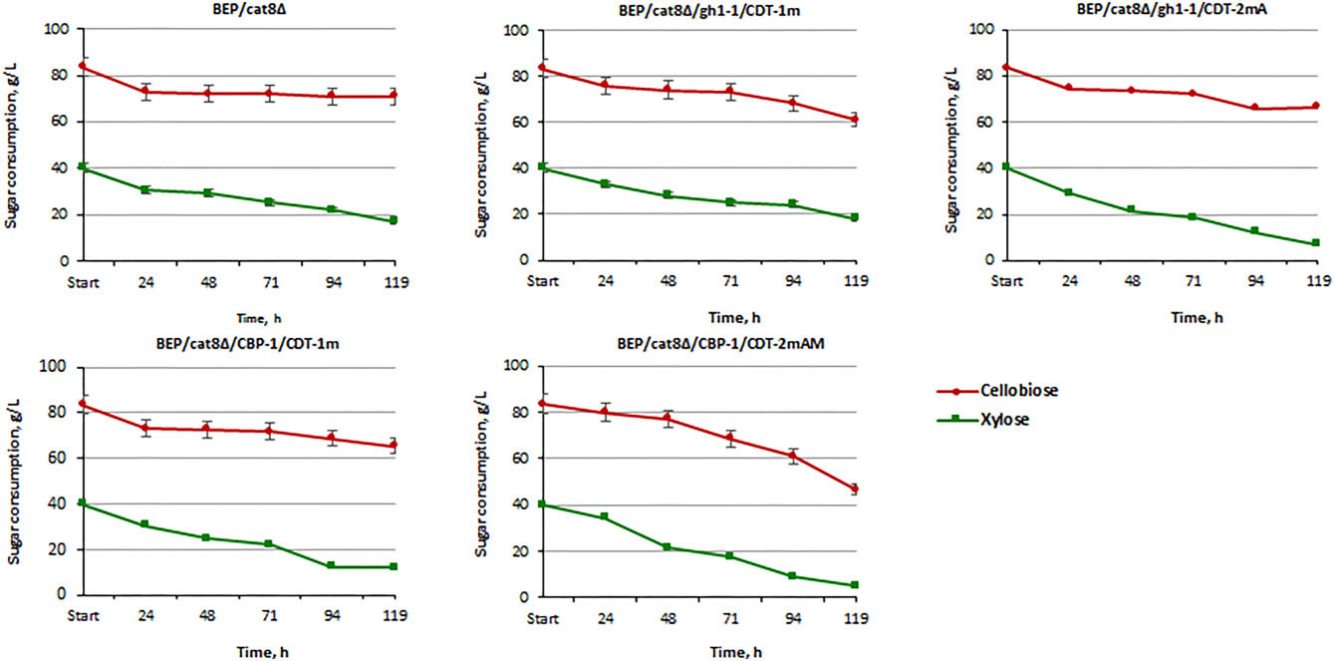
Cellobiose and xylose consumption by the parental strain BEP/cat8Δ, evolutionary mutants BEP/cat8∆/gh1-1/CDT-2mA, BEP/cat8∆/CBP-1/CDT-2mAM, and obtained strains BEP/cat8∆/gh1-1/CDT-1m, BEP/cat8∆/CBP-1/CDT-1m during alcoholic fermentation at 45°C in the media with 8% cellobiose/4% xylose. «A»—mutants obtained by laboratory evolution, «AM»—mutants obtained in two stages as a result of laboratory evolution, and UV—mutagenesis with 2-DG as a glycolysis inhibitor.

We hypothesized that the repression of xylose utilization by glucose could be alleviated in the obtained recombinant and mutant strains, due to intracellular hydrolysis of cellobiose. As a result of intracellular hydrolysis, the competition between glucose and xylose for transporters will be reduced, and the level of glucose-dependent repression will be diminished. This will enable yeast to more efficiently utilize xylose, even in the presence of glucose. The profiles of sugar consumption and ethanol production by *O. polymorpha* strains with overexpressed gene pairs gh1-1/CDT-1m and CBP-1/CDT-1m, as well as strains with the overexpression of gh1-1/CDT-2mA and CBP-1/CDT-2mAM after selective screening, were compared (Fig. 7). During 119 h of 8% cellobiose/4% xylose cofermentation the BEP/*cat8∆*/CBP-1/CDT-2mAM consumed 90% of xylose and 45% of cellobiose. Thus, the adapted strain selected under selective conditions after UV irradiation BEP/cat8∆/CBP-1/CDT-2mAM exhibited the best sugar consumption parameters for both sugars (Fig. 7). The BEP/cat8∆/gh1-1/CDT-1m, BEP/cat8∆/CBP-1/CDT-1m, or BEP/cat8∆/gh1-1/CDT-2mA strains exhibited a modest consumption rate of both sugars. The ethanol production level during cofermentation by BEP/cat8∆/CBP-1/CDT-2mAM strain reached 7 g/l at 71 h of fermentation, representing the highest among the analyzed strains (Fig. 9A). However, it is worth noting the potential synergistic effect of cofermentation, as fermentation of the mixture gave a higher level of ethanol production compared to 10% xylose or 10% cellobiose fermentation separately. These results suggest that cofermentation of cellobiose and xylose can enhance overall ethanol yield and productivity (Fig. 9A, C, and D).

**Figure 8.**
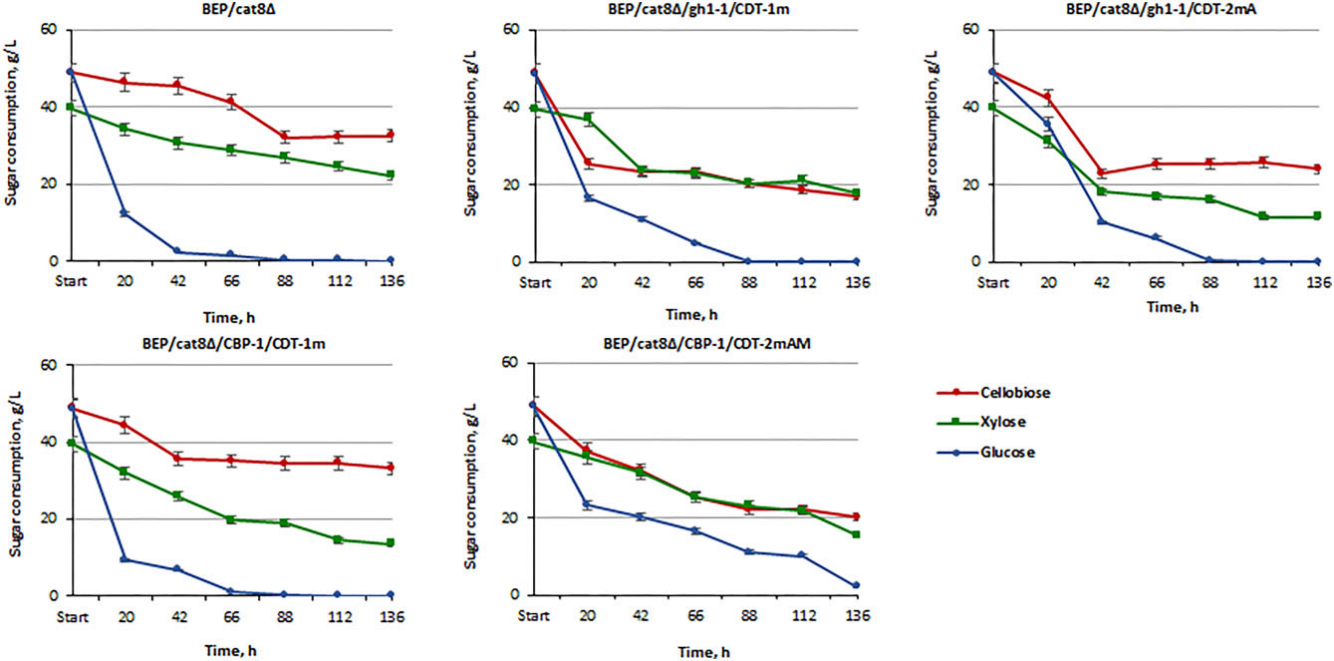
Cellobiose, glucose, and xylose consumption by the parental strain BEP/cat8Δ, evolutionary mutants BEP/cat8∆/gh1-1/CDT-2mA, BEP/cat8∆/CBP-1/CDT-2mAM, and obtained strains BEP/cat8∆/gh1-1/CDT-1m, BEP/cat8∆/CBP-1/CDT-1m during alcoholic fermentation at 45°C in the media with 5% cellobiose/4% xylose/5% glucose. «A»—mutants obtained by laboratory evolution, «AM»—mutants obtained in two stages as a result of laboratory evolution, and UV—mutagenesis with 2-DG as a glycolysis inhibitor.

**Figure 9.**
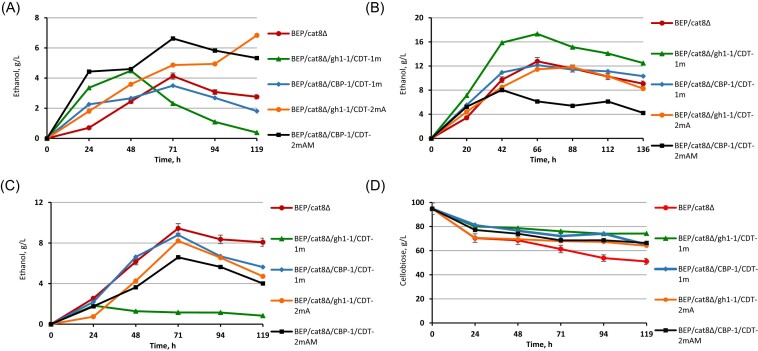
Ethanol production by the parental strain BEP/cat8Δ, evolutionary mutants BEP/cat8∆/gh1-1/CDT-2mA, BEP/cat8∆/CBP-1/CDT-2mAM, and obtained strains BEP/cat8∆/gh1-1/CDT-1m, BEP/cat8∆/CBP-1/CDT-1m during alcoholic fermentation at 45°C in the media with different sugars ratio (A) 8% cellobiose/4% xylose, (B) 5% cellobiose/4% xylose/5% glucose. Ethanol production (C) and xylose consumption (D) by the parental strain BEP/cat8Δ, evolutionary mutants BEP/cat8∆/gh1-1/CDT-2mA, BEP/cat8∆/CBP-1/CDT-2mAM, and obtained strains BEP/cat8∆/gh1-1/CDT-1m, BEP/cat8∆/CBP-1/CDT-1 m during alcoholic fermentation at 45°C in the media with 10% xylose. «A»—mutants obtained by laboratory evolution, «AM»—mutants obtained in two stages as a result of laboratory evolution, and UV—mutagenesis with 2-DG as a glycolysis inhibitor.

Within 42 h of cofermentation with 5% cellobiose, 4% xylose, and 5% glucose, the best xylose-fermenting strains (BEP/cat8∆) utilized 98% of glucose, while a significant amount of xylose and cellobiose remained in the medium (Fig. 8). The BEP/cat8∆/gh1-1/CDT-1m and BEP/cat8∆/CBP-1/CDT-1m strains exhibited a decrease in all sugars by the 42 h of fermentation, but later showed a preference for glucose. The BEP/cat8∆/gh1-1/CDT-2mA strain displayed a similar sugar consumption dynamic to BEP/cat8∆/gh1-1/CDT-1m; however, accelerated depletion of glucose, xylose, and even cellobiose within the first 42 h of fermentation allowed for the utilization of over 60% of xylose, while more than 50% of cellobiose remained even after 136 h of fermentation. The BEP/cat8∆/CBP-1/CDT-2mAM strains slowly and simultaneously utilized all sugars; however, 50% of xylose and 50% of cellobiose still persisted in the medium after 136 h of fermentation. These results indicate that the expression of different gene combinations in the strains influenced their sugar utilization patterns, with some strains demonstrating an early preference for glucose consumption, while others efficiently utilized glucose, xylose, and cellobiose over time. It is also worth noting that strains BEP/cat8∆/gh1-1/CDT-2mA and BEP/cat8∆/CBP-1/CDT-2mAM were obtained through adaptation and mutagenesis processes, which may have led to the accumulation of additional genetic changes that positively influenced sugar metabolism. Based on our previous knowledge of xylose transporters (Vasylyshyn et al. 2020), we hypothesized that additional native xylose transporters might interfere with heterologous cellobiose transport. Moreover, xylose transporters are inhibited by the presence of glucose, which has led to many efforts to relieve this inhibition in *S. cerevisiae* (Farwick et al. 2014, Nijland et al. 2014). Thus, xylose transport in the parental strain occurred after complete glucose utilization. In the BEP/cat8∆/gh1-1/CDT-1m and BEP/cat8∆/CBP-1/CDT-1m strains, a synergistic effect of xylose and cellobiose was observed, but glucose still remained preferred. Only the BEP/cat8∆/CBP-1/CDT-2mAM strain utilized all sugars simultaneously, albeit with low speed and low ethanol yield (Fig. 9B). However, it is also worth noting that glucose consumption is impaired in all modified strains compared to the parental strain. Considering the fact that monogenic overexpression of the transporters was not characterized by a significant use of cellobiose (Figure S3, Supporting Information), these results indicate that the introduction of heterologous transporters CDT-1m and CDT-2m is not decisive for cellobiose utilization but plays a role in its transport in *O. polymorpha*.

## Discussion

In this research, we started to solve a notable technical obstacle that impedes the cost-effective generation of cellulosic biofuels. This obstacle involves the constrained capability of fermenting microorganisms to effectively utilize various carbon components derived from lignocellulosic biomass. To overcome this hurdle, we applied metabolic engineering, laboratory evolution, and mutagenesis methods to incorporate enhanced fermentation pathways for cellobiose in yeast with improved xylose fermentation parameters of *O. polymorpha* BEP/cat8∆ strain.

Wild-type *O. polymorpha* NCYC495 cannot assimilate cellobiose. Therefore, it is crucial to introduce genes encoding cellobiose transporters (CDTs) and intracellular enzymes (ß-glucosidase or CBP) that hydrolyze cellobiose, the components of the heterologous cellobiose metabolic pathway (Galazka et al. 2010, Ha et al. 2013, Kim et al. 2014). Direct fermentation of cellodextrins instead of glucose is advantageous because glucose inhibits cellulases activity and represses the fermentation of xylose present in cellulosic hydrolysates. The enzyme β-glucosidase that converts cellobiose and soluble cellodextrins to glucose has been shown to be one of the major rate-limiting steps in the saccharification of cellulose (Lynd et al. 2002). However, it requires two moles of ATP to initiate glycolysis (Galazka et al. 2010, Kim et al. 2014). CBP is an energy-efficient enzyme (using only 1 molecule of ATP) (Ha et al. 2013, Choi et al. 2022a) capable of hydrolyzing cellobiose to glucose and glucose-1-phosphate (G1P) in the presence of inorganic phosphate. Unfortunately, its activity is significantly reduced in the presence of xylose. Xylose is a known mixed inhibitor of CBP enzyme, decreasing CBP's apparent affinity for cellobiose and reducing its apparent maximum velocity (Chomvong et al. 2017a). Moreover, two CDTs (CDT-1 and CDT-2) were previously identified in *N. crassa*, but their kinetic properties and efficiency for cellobiose fermentation of other yeasts have not been studied in detail (Cai et al. 2014).

Due to the introduction of the mentioned modified transport systems and heterologous intracellular metabolic pathways, we have successfully created *O. polymorpha* yeast strains BEP/cat8∆/gh1-1/CDT-1m and BEP/cat8∆/CBP-1/CDT-1m with improved parameters for high-temperature (45°C) alcoholic fermentation of cellobiose, while strains containing the CDT-2m transporter did not metabolize this sugar. However, the biomass accumulation rate in the obtained transformants BEP/cat8∆/gh1-1/CDT-2m and BEP/cat8∆/CBP-1/CDT-2m was four times higher than that of the parental strain. Paradoxically, BEP/cat8∆/gh1-1/CDT-1m practically did not accumulate biomass under conditions of sufficient aeration but successfully fermented cellobiose under fermentation conditions, unlike BEP/cat8∆/CBP-1/CDT-1m. The inferior growth parameters of the BEP/cat8∆/gh1-1/CDT-1m strain may be associated with its overall higher energy expenditure (3 ATP molecules) compared to BEP/cat8∆/CBP-1/CDT-1m (2 ATP molecules). Moreover, the cellobiose utilization system used here does not generate extracellular glucose, which acts as an important signaling molecule for yeast carbon metabolism (Lin et al. 2014, Chomvong et al. 2017b). This could be the reason for the low ethanol yields observed in this study and it warrants further investigation in this direction to enhance the performance of *O. polymorpha* yeast.

*Neurospora crassa* CDT-1 m and CDT-2 m belong to the same transporter family as the HXT transporters (Transporter Classification Database identifier 2.A.1.1; http://www.tcdb.org) (Saier et al. 2014). Thus, downregulation of CDT-1m and CDT-2m might remove them from the cell surface, thereby imposing a limitation on the efficacy of cellobiose utilization and ethanol production from this carbon source. The endocytosis of glucose transporters Hxt1 and Hxt3 can be stimulated by adding 2-DG (O’Donnell et al. 2015), a cytotoxic analog of glucose, to the medium, resulting in mutants with altered transporter properties. We tried to adapt yeast with *gh1-1/CDT-2m* and *CBP-1/CDT-2m* gene combinations for cellobiose consumption. The yeast BEP/cat8∆/gh1-1/CDT-2m and BEP/cat8∆/CBP-1/CDT-2m quickly adapted, and the use of UV mutagenesis and 2-DG allowed the obtaining of BEP/cat8∆/CBP-1/CDT-2mAM mutants with the ethanol production level from 10% cellobiose increased 4-fold (Figs 3B and 5C). This approach represents an innovative strategy that combines metabolic engineering, laboratory evolution, and mutagenesis, enabling the integration or activation of numerous substrate utilization pathways to enhance biocatalytic conversion. Therefore, evolution engineering was an efficient approach to improve the cellobiose utilization of the engineered yeast strain.

The ethanol yield during fermentation of 10% cellobiose by the yeast strain without adaptive changes, BEP/cat8∆/gh1-1/CDT-1m, and the strain BEP/cat8∆/gh1-1/CDT-2mA (obtained through adaptive evolution) was very similar (Table 1). Therefore, we hypothesized that BEP/cat8∆/gh1-1/CDT-2mA, which ferments cellobiose, could demonstrate equivalent efficiency during cofermentation of xylose and cellobiose. During the coutilization of 8% cellobiose and 4% xylose (Fig. 7), recombinant strains BEP/cat8∆/gh1-1/CDT-1m, BEP/cat8∆/gh1-1/CDT-2mA, BEP/cat8∆/CBP-1/CDT-1m, and the parental strain BEP/cat8∆ exhibited a preference for xylose. Furthermore, the differential utilization of sugars observed among these strains highlights the complexity of metabolic pathways involved in mixed sugar utilization and underscores the need for further metabolic engineering strategies. It should be noted that the introduction of several heterologous pathways into one microorganism could also lead to a harmful metabolic load, especially at high sugar concentrations (Bobadilla Fazzini et al. 2010). However, the BEP/cat8∆/CBP-1/CDT-2mAM strain actively utilized cellobiose, resulting in significantly more efficient xylose uptake and the high level of ethanol production compared to the other engineered strains obtained.

**Table 1.** Main parameters of cellobiose fermentation at 45°C by the tested *O. polymorpha* strains with *gh1-1/CDT-1m, CBP-1/CDT-1m, gh1-1/CDT-2mA*, and *CBP-1/CDT-2mAM* simultaneous overexpression genes. «A»—mutants obtained by laboratory evolution, «AМ»—mutants obtained in two stages as a result of laboratory evolution, and UV—mutagenesis with 2-DG as a glycolysis inhibitor.

Strain	Ethanol (g/l)	Ethanol yield (g/g consumed cellobiose)	Ethanol-specific production rate (g/g biomass/h) dry weight	Ethanol productivity (g/l/h)
BEP/cat8∆^b^	0.47 ± 0.032	0.041 ± 0.002	0.004 ± 0.001	0.007 ± 0.001
BEP/cat8∆/gh1-1/CDT-1m^c^	4.60 ± 0.102	0.180 ± 0.028	0.027 ± 0.009	0.076 ± 0.011
BEP/cat8∆/CBP-1/CDT-1m ^c^	0.79 ± 0.041	0.045 ± 0.004	0.006 ± 0.002	0.013 ± 0.003
BEP/cat8∆/gh1-1/CDT-2mA ^c^	0.68 ± 0.062	0.047 ± 0.004	0.005 ± 0.001	0.011 ± 0.001
BEP/cat8∆/CBP-1/CDT-2mAM^a^	4.20 ± 0.108	0.183 ± 0.031	0.025 ± 0.010	0.070 ± 0.009

a Data of ethanol yield and ethanol (g/l) are represented on YNB medium supplemented with 10% of cellobiose on 48 h of fermentation.

b 72 h of fermentation.

c 96 h of fermentation.

During cofermentation of 5% cellobiose/4% xylose/5% glucose (Fig. 8), we expected that the presence of small amounts of glucose that can be formed as a result of pretreatment and hydrolysis of lignocellulosic materials would not affect the ability of engineered yeast to convert sugar mixtures of hexoses and pentoses into ethanol. The presence of glucose significantly altered the perception and expected outcome of alcohol fermentation. Strains BEP/cat8∆/gh1-1/CDT-1m, BEP/cat8∆/gh1-1/CDT-2mA, which initially utilized only 25% of the cellobiose during cofermentation of both sugars, began actively consuming it, but this process ceased after complete depletion of glucose. Xylose utilization was also severely hindered. Strain BEP/cat8∆/CBP-1/CDT-1m did not show a significant reduction in cellobiose consumption but continued to use xylose, even after complete depletion of glucose. In contrast, strain BEP/cat8∆/CBP-1/CDT-2mAM achieved complete glucose utilization only at 136 h of fermentation while simultaneously exhibiting low-intensity consumption of xylose and cellobiose. Surprisingly, BEP/cat8∆/CBP-1/CDT-2mAM strain accumulated only 8 g/l of ethanol despite the simultaneous and uniform utilization of all sugars, while strains BEP/cat8∆/gh1-1/CDT-2 mA and BEP/cat8∆/CBP-1/CDT-1m exhibited similar ethanol production levels, but drastically different sugar consumption rates (Fig. 9). Their ethanol production levels reached 12 g/l, proportional to the parental strain, indicating that, for some reason, the amount of consumed cellobiose did not influence the ethanol yield in these variants. Here, we highlight the observed negative impact of extracellular glucose on the CDT-2mAM transporter. It is important to note that, in this study, we did not determine the transport activity directly. Therefore, a more detailed assessment of the transporter’s functionality is required for a comprehensive understanding. Furthermore, CBP, in the presence of xylose, can also lose its affinity for cellobiose, and xylose and glucose-1-phosphate can be used as substrates for the reverse reaction with CBP, leading to the formation of a side products (Chomvong et al. 2017b). Thus, yeast strains with the double block BEP/cat8∆/CBP-1/CDT-2mAM exhibited the lowest level of ethanol production and impaired glucose uptake in the cofermentation environment. Yeast strains in which one of the systems, either CDT-2 m or CBP, was blocked, were able to use glucose and possibly, in the case of BEP/cat8∆/CBP-1/CDT-1 m, xylose, similar to the parental strain. Strain BEP/cat8∆/gh1-1/CDT-1m was not affected by negative regulation by glucose or xylose in the environment, resulting in an ethanol production level of 17.5 g/l (Fig. 9). It is worth noting that the CDT-2mAM transporter has undergone multiple changes, and the theoretical effect of reverting to the original state could be induced by glucose. To challenge this idea, cells were collected after cofermentation with glucose and used for monofermentation with 10% cellobiose. Ethanol production reached 4 g/l (data not shown), consistent with previous results (Fig. 5C).

Today, a number of microorganisms are known to have a natural or acquired ability to ferment cellobiose into ethanol. For example, *S. cerevisiae*, which accumulates 38 g/l of ethanol at 30°C (Choi et al. 2022), *Myceliophthora thermophila—*11.3 g/l of ethanol at 45–50°C, (Li et al. 2020), *Zymobacter palmae*—10 g/l of ethanol at 30°C, (Yanase et al. 2005). The *O. polymorpha* currently produces a maximum of only 5 g/l of ethanol at 45°C. Despite the incomplete utilization of cellobiose and the low ethanol yield, which undoubtedly require further investigations into the fermentative activity and regulatory mechanisms involved in cellobiose metabolism, we have succeeded in obtaining yeast capable of simultaneous consumption of all quantitatively significant sugars in lignocellulose hydrolysates. To date, this is the first report of the successful development of stable methylotrophic thermotolerant strains of *O. polymorpha* capable of efficiently coutilizing cellobiose, glucose, and xylose under high-temperature alcoholic fermentation conditions at 45°C. We suggest that further improvement of cellobiose utilization and fermentation by the constructed strain could be possible due to multicopy integration of genes coding for cellobiose transport and hydrolysis. Alternative (or/and additional) approachs could be based on selection of the mutants resistant to growth inhibition on cellobiose by 3-bromopyruvate (Kurylenko et al. 2018) or other inhibitors (Dmytruk et al. 2016).

## Supplementary Material

foae007_Supplemental_File
