# Supplementary material for: Engineering of Ogataea polymorpha strains with ability for high-temperature alcoholic fermentation of cellobiose
Source: FEMS Yeast Res. 2024 Feb 23;24:foae007. doi: 10.1093/femsyr/foae007 (PMC10929770; doi:10.1093/femsyr/foae007)
Supplement: foae007_Supplemental_File [file foae007_supplemental_file.pdf]

Supporting informations

FEMS Yeast Research

ENGINEERING OF *OGATAEA POLYMORPHA* STRAINS WITH ABILITY FOR HIGH-TEMPERATURE ALCOHOLIC FERMENTATION OF CELLOBIOSE

Roksolana Vasylyshyn[1,2], Olena Dmytruk[1,2], Andriy Sibirny[1,2], Justyna Ruchala[1,2*]

[1] University of Rzeszow, College of Natural Sciences, Institute of Biotechnology

[2] Institute of Cell Biology NAN of Ukraine, Department of Molecular Genetics and Biotechnology

*Corresponding author: jruchala@ur.edu.pl

Table S1 Sequence *gh1-1* gene from *Neurospora crassa, CBP* gene from *Saccharophagus degradans*, *CDT-1m* gene from *N. crassa, CDT-2m* gene from *N. crassa* optimized for *Ogataea polymorpha.*

>gh1-1 *N. crassa* optimised for *O. polymorpha*
TCTAGAATGTCTCTGCCAAAAGATTTTCTGTGGGGCTTTGCCACCGCCGCCTATCAGATTGAAGGCGCCATTCACGCCGAT
GGCAGAGGCCCATCTATTTGGGATACCTTTTGCAACATTCCAGGCAAAATTGCCGATGGCTCTTCTGGCGCCGTGGCCTGC
GATTCTTATAACAGAACCAAAGAAGATATTGATCTGCTGAAATCTCTGGGCGCCACCGCCTATAGATTTTCTATTTCTTGG
TCCAGAATTATTCCAGTGGGCGGCAGAAACGATCCAATTAACCAGAAAGGCATTGATCACTATGTGAAATTTGTGGATGAT
CTGCTGGAAGCCGGCATTACCCCATTTATTACCCTGTTTCACTGGGATCTGCCAGATGGCCTGGATAAAGATATGGCGGC
CTGCTGAACAGAGAAGAATTTCCACTGGATTTTGAACACTATGCCAGAACCATGTTTAAAGCCATTCCAAAATGCAAACAC
TGGATTACCTTTAACGAACCATGGTGCTCTTCTATTCTGGGCTATAACTCTGGCTATTTTGCCCCAGGCCACACCTCTGAT
AGAACCAAATCTCCAGTGGGCGATTCTGCCAGAGAACCATGGATTGTGGGCCACAACCTGCTGATTGCCCACGGCAGAGCC
GTGAAAGTGTATAGAGAAGATTTTAAACCAACCCAGGGCGGCGAAATTGGCATTACCCTGAACGGCGATGCCACCCTGCCA
TGGGATCCAGAAGATCCACTGGATGTGGAAGCCTGCGATAGAAAAATTGAATTTGCCATTTCTTGGTTTGCCGATCCAATT
TATTTTGGCAAATATCCAGATTCTATGAGAAAACAGCTGGGCGATAGACTGCCAGAATTTACCCCAGAAGAAGTGGCCCTG
GTGAAAGGCTCTAACGATTTTTATGGCATGAACCACTATACCGCCAACTATATTAAACACAAAAAAGGCGTGCCACCAGAA
GATGATTTTCTGGGCAACCTGGAAACCCTGTTTTATAACAAAAAAGGCAACTGCATTGGCCCAGAAACCCAGTCTTTTTGG
CTGAGACCACACGCCCAGGGCTTTAGAGATCTGCTGAACTGGCTGTCTAAAAGATATGGCTATCCAAAAATTTATGTGACC
GAAAACGGCACCTCTCTGAAAGGCGAAAACGCCATGCCACTGAAACAGATTGTGGAAGATGATTTTAGAGTGAAATATTTT
AACGATTATGTGAACGCCATGGCCAAAGCCCACTCTGAAGATGGCGTGAACGTGAAAGGCTATCTGGCCTGGTCTCTGATG
GATAACTTTGAATGGGCCGAAGGCTATGAAACCAGATTTGGCGTGACCTATGTGGATTATGAAAACGATCAGAAAAGATAT
CCAAAAAAATCTGCCAAATCTCTGAAACCACTGTTTGATTCTCTGATTAAAAAAGATTAAGCGGCCGC

>CBP *S. degradans* optimized for *O. polymorpha*
TCTAGA**ATG**AAATTTGGCCACTTTGATGATAAAGCCAGAGAATATGTGATTACCGATCCAAAAACCCCATATCCATGGATT
AACTATCTGGGCAACGAAGATTTTTTTCGCTGGTGTCGAACACCGGCGGCGGCTATTCGTTTTATAAAGATGCCAAATTT
AGAAGACTGACCAGATATAGATATAACAACGTGCCAGTGGATAACGGCGGCAAATATTTTTATATTAACGATTCGGGCGAT
GTGTGGTCGCCAGGCTGGAAACCAGTGAAAGCCGAACTGGATGCCTATTCGTGCGCCCACGGCCTGTCGTATACCAGAATT
ACCGGCGAAAGAAACGGCATTCAGGCCGAAGTGCTGTCGTTTATTCCACTGGGCACCTGGGCCGAAATTCAGAAAGTGTCG
CTGAAAAACACCTCGGGCGCCACCAAAAAATTTAAACTGTTTTCGTTTGCCGAATGGTGCCTGTGGAACGCCGAAGATGAT
ATGACCAACTTTCAGAGAAACTTTTCGACCGGCGAAGTGGAAGTGGAAGATTCGGTGATTTATCACAAAACCGAATTTAAA
GAAAGAAGAAACCACTATGCCTTTTATTCGGTGAACGCCCCAATTCAGGGCTTTGATACCGATAGAGATAAATGGAAAGGC
CTGTATAACGATTTTGATAAACCAGATGCCGTGTTTGAAGGCGAACCAAGAAACTCGGAAGCCCACGGCTGGTCGCCAATT
GCCTCGCACTATCTGGAAGTGGAACTGGCCCCAGGCGAATCGAAAGATCTGATTTTTGTGCTGGGCTATATTGAAGTGGCC
CCAGAAAACAAATGGGAATCGAAAGGCGTGATTAACAAATCGCCAGCCAAAGAACTGATTGCCAGATTTGATTCGGTGGAA
AAAGTGGATGCCGAACTGACCAAACTGGCCGATTATTGGGCCAACCTGCTGTCCACCTATTCGGTGGAATCGGGCGATGAA

AAACTGGATAGAATGGTGAACATTTGGAACCAGTATCAGTGCATGGTGACCTTTAACATGTCGAGATCGGCCTCGTTTTTT
GAATCGGGCATTGGCAGAGGCATGGGCTTTAGAGATTCGAACCAGGATCTGATTGGCTTTGTGCACCAGGTGCCAGAAAGA
GCCAGAGAAAGAATTATTGATATTGCCTCGACCCAGTTTGAAGATGGCTCGGCCTATCACCAGTATCAGCCACTGACCAAA
AGAGGCAACAACGCCATTGGCGGCAACTTTAACGATGATCCACTGTGGCTGATTCTGTCCACCACCGATTATATTAAAGAA
ACCGGCGATTTTTCGATTCTGGAAGAACAGGTGCCATATGATAACGATGCCTCGAAAGCCACCTCGCACTTTGAACACCTG
AAAAGATCGTTTTATCACACCGTGAACAACCTGGGCCCACACGGCCTGCCACTGATTGGCAGAGCCGATTGGAACGATTGC
CTGAACCTGAACTGCTTTTCGGAAGATCCAAACGAATCGTTTCAGACCACCGGCAACAAAACCGGCAGAACCGCCGAATCG
CTGATGATTGCCGGCCTGTTTGTGCTGTATGGCAACGAATTTGTGAAACTGTGCAGAGAAATTGGCCAGGATGGCGAAGCC
GCCGAAGCCCAGGCCCACATTGATCAGATGGTGGAAGCCGTGAAAAAACACGGCTGGGATGGCGAATGGTTTCTGAGAGCC
TATGATTATTATGGCAAAAAAGTGGGCTCGAAAGAAAACGAAGAAGGCAAAATTTTTATTGAATCGCAGGGCTTTTGCGGC
ATGGCCGGCATTGGCCTGGAAGATGGCCTGGTGGAAAAATCGATGGATTCGGTGAAAGAATGGCTGGATTGCGATTATGGC
ATTGTGCTGCAGCAGCCAGCCTTTACCAAATATTATATTGAATATGGCGAAATTTCGACCTATCCAGCCGGCTATAAAGAA
AACGCCGGCATTTTTTGCCACAACAACCCATGGATTATGATTACCGAAACCCTGCTGGGCAGAGGCGATAAAGCCTTTGAA
TATTATAGAAAAATTGCCCCAGCCTATCTGGAAGAAATTTCGGATCTGCACAAAGTGGAACCATATGCCTATTGCCAGATG
ATTGCCGGCAAAGATGCCTATCTGCCAGGCGAAGGCAAAAACTCGTGGCTGACCGGCACCGCCTCGTGGAACTTTGCCGCC
ATTACCCAGTATATTCTGGGCGTGAAACCAGATTATTCGGGCCTGGCCATTAACCCATGCATTCCATCGTCGTGGGATGGC
TTTAAAGTGACCAGAAAATATAGAGGCGCCACCTATAACATTATTGTGACCAACCCAACCCACGTGTCGAAAGGCGTGAAA
TCGCTGACCCTGAACGGCAACGCCATTGATGGCTATATTGTGCCACCACAGCAGGCCGGCACCGTGTGCAACGTGGAAGTG
ACCCTGGGC**TAA**GCGGCCGC

>CDT-1m *N. crassa* optimized for *O. polymorpha*
TCTAGA**ATG**TCTTCTCACGGCTCTCACGATGGCGCTTCTACCGAAAAACACCTGGCTACCCACGATATTGCTCCAACCCAC
GATGCTATTAAAATTGTGCCAAAAGGCCACGGCCAGACCGCTACCAAACCAGGCGCTCAGGAAAAAGAAGTGAGAAACGCT
GCTCTGTTTGCTGCTATTAAAGAATCTAACATTAAACCATGGTCTAAAGAATCTATTCACCTGTACTTTGCTATTTTTGTG
GCTTTTTGCTGCGCTTGCGCTAACGGCTACGATGGCTCTCTGATGACCGGCATTATTGCTATGGATAAATTTCAGAACCAG
TTTCACACCGGCGATACCGGCCCAAAAGTGTCTGTGATTTTTCTCTGTACACCGTGGGCGCTATGGTGGGCGCTCCATTT
GCTGCTATTCTGTCTGATAGATTTGGCAGAAAAAAAGGCATGTTTATTGGCGGCATTTTTATTATTGTGGGCTCTATTATT
GTGGCTTCTTCTTCTAAACTGGCTCAGTTTGTGGTGGGCAGATTTGTGCTGGGCCTGGGCATTGCTATTATGACCGTGGCT
GCTCCAGCTTACTCTATTGAAATTGCTCCACCACACTGGAGAGGCAGATGCACCGGCTTTTACAACTGCGGCTGGTTGGGC
GGCTCTATTCCAGCTGCTTGCATTACCTACGGCTGCTACTTTATTAAATCTAACTGGTCTTGGAGAATTCCACTGATTCTG
CAGGCTTTTACCTGCCTGATTGTGATGTCTTCTGTGTTTTTTCTGCCAGAATCTCCAAGATTTCTGTTTGCTAACGGCAGA
GATGCTGAAGCTGTGGCTTTTCTGGTGAAATACCACGGCAACGGCGATCCAAACTCTAAACTGGTGCTGCTGGAAACCGAA
GAAATGAGAGATGGCATTAGAACCGATGGCGTGGATAAAGTGTGGTGGGATTACAGACCACTGTTTATGACCCACTCTGGC
AGATGGAGAATGGCTCAGGTGCTGATGATTTCTATTTTTGGCCAGTTTTCTGGCAACGGCCTGGGCTACTTTAACACCGTG
ATTTTTAAAAACATTGGCGTGACCTCTACCTCTCAGCAGCTGGCTTACAACATTCTGAACTCTGTGATTTCTGCTATTGGC
GCTCTGACCGCTGTGTCTATGACCGATAGAATGCCAAGAAGAGCTGTGCTGATTATTGGCACCTTTATGTGCGCTGCTGCT
CTGGCTACCAACTCTGGCCTGTCTGCTACCCTGGATAAACAGACCCAGAGAGGCACCCAGATTAACCTGAACCAGGGCATG
AACGAACAGGATGCTAAAGATAACGCTTACCTGCACGTGGATTCTAACTACGCTAAAGGCGCTCTGGCTGCTTACTTTCTG
TTTAACGTGATTTTTTCTTTTACCTACACCCCACTGCAGGGCGTGATTCCAACCGAAGCTCTGGAAACCACCATTAGAGGC
AAAGGCTGGCTCTGTCTGGCTTTATTGTGAACGCTATGGGCTTTATTAACCAGTTTGCTGGCCCAATTGCTCTGCACAACA
TTGGCTACAAATACATTTTTGTGTTTGTGGGCTGGGATCTGATTGAAACCGTGGCTTGGTACTTTTTTGGCGTGGAATCTC
AGGGCAGAACCCTGGAACAGCTGGAATGGGTGTACGATCAGCCAAACCCAGTGAAAGCTTCTCTGAAAGTGGAAAAAGTGG
TGGTGCAGGCTGATGGCCACGTGTCTGAAGCTATTGTGGCT**TAA**GCGGCCGC

>CDT-2m *N. crassa* optimized for *O. polymorpha*
TCTAGA**ATG**GGCATTTTTAACAAAAAACCAGTGGCCCAGGCCGTGGATCTGAACCAGATTCAGGAAGAAGCCCCACAGTTT
GAAAGAGTGGATTGGAAAAAAGATCCAGGCCTGAGAAAACTGTACTTTTACGCCTTTATTCTGTGCATTGCCTCGGCCACC
ACCGGCTACGATGGCATGTTTTTTAACTCGGTGCAGAACTTTGAAACCTGGATTAAATACTTTGGCGATCCAAGAGGCTCG
GAACTGGGCCTGCTGGGCGCCCTGTACCAGATTGGCTCGATTGGCTCGATTCCATTTGTGCCACTGCTGACCGATAACTTT
GGCAGAAAAACCCCAATTATTATTGGCTGCGTGATTATGATTGTGGGCGCCGTGCTGCAGGCCACCGCCAAAAACCTGGAT
ACCTTTATGGGCGGCAGAACCATGCTGGGCTTTGGCAACTCGCTGGCCCAGATTGCCTCGCCAATGCTGCTGACCGAACTG
GCCCACCCACAGCACAGAGCCAGACTGACCACCATTTACAACTGCCTGTGGAACGTGGGCGCCCTGGTGGTGTCGTGGCTG
GCCTTTGGCACCAACTACATTAACAACGATTGGTCGTGGAGAATTCCAGCCCTGCTGCAGGCCTTTCCATCGATTATTCAG
CTGCTGGGCATTTGGTGGGTGCCAGAATCGCCAAGATTTCTGATTGCCAAAGATAAACACGATGAAGCCCTGCACATTCTG
GCCAAATACCACGCCAACGGCGATCCAAACCACCCAACCGTGCAGTTTGAATTTAGAGAAATTAAAGAAACCATTAGACTG
GAAATGGAATCGACCAAAAACTCGTCGTACCTGGATTTTTTTAAATCGAGAGGCAACAGATACAGACTGGCCATTCTGCTG
TCGCTGGGCTTTTTTTCGCAGTGGTCGGGCATCGCCATTATTTCGAACTACTCGTCGAAACTGTACGAAACCGCCGGCGTG
ACCGATTCGACCGCCAAACTGGGCCTGTCGGCCGGCCAGACCGGCCTGGCCCTGATTGTGTCGGTGACCATGGCCCTGCTG
GTGGATAAACTGGGCAGAAGACTGGCCTTTCTGGCCTCGACCGGCGGCATGTGCGGCACCTTTGTGATTTGGACCCTGACC

GCCGGCCTGTACGGCGAACACAGACTGAAAGGCGCCGATAAAGCCATGATTTTTTTTATTTGGGTGTTTGGCATTTTTTAC
TCGCTGGCCTGGTCGGGCCTGCTGGTGGGCTACGCCATTGAAATTCTGCCATACAGACTGAGAGGCAAAGGCCTGATGGTG
ATGAACATGTCGGTGCAGTGCGCCCTGACCCTGAACACCTACGCCAACCCAGTGGCCTTTGATTACTTTGGCCCAGATCAC
TCGTGGAAACTGTACCTGATTTACACCTGCTGGATTGCCGCCGAATTTGTGTTTGTGTTTTTTATGTACGTGGAAACCAAA
GGCCCAACCCTGGAAGAACTGGCCAAAGTGATTGATGGCGATGAAGCCGATGTGGCCCACATTGATATTCACCAGGTGGAA
AAAGAAGTGGAAATTCACGAACACGAAGGCAAATCGGTGGCC**TAA**GCGGCCGC

Table S2 List of primers used in this study

**Primer name**	**Primer sequence 5’-3’†**
Ko1224	AGTCATACGTGTAGGTTTTTGGC
Ko1237	GTTAATTGGATCGTTTCTGCCG
Ko1238	CAGTGGAATAAACGACAGCAC
Ko1239	GTATCGCCGGTGTGAAACTG
Ko1240	CCACAATCATAATCACGCAGC
Ko1328	CCCGAGCTCTAGACCACATCCGTGCACCAGACAAG
Ko1329	CGCGGATCCCTGCCACGAGGTACCACAAAGATTATAAAG
Ko1330	CGCGTCGACTAGACCACATCCGTGCACCAGACAAG
Ko1331	CCCAAGCTTCTGCCACGAGGTACCACAAAGATTATAAAG
Ko1332	CGCGTCGACCTGCCACGAGGTACCACAAAGATTATAAAG

Fig. S1 Confirmation of the correctness of joint overexpression of genes *gh1-1/CDT-1* (3, 4, 8, 9), *CBP-1/CDT-1* (19.6, 54.2, 6.2, 37.1*)*, *gh1-1/CDT-2* (5, 8, 19, 25, 30), and *CBP-1/CDT-2* (3, 4, 16, 21, 24) in the genome of the BEP/*cat8*Δ strain by means of PCR using pairs of marked primers; L – molecular weight marker.

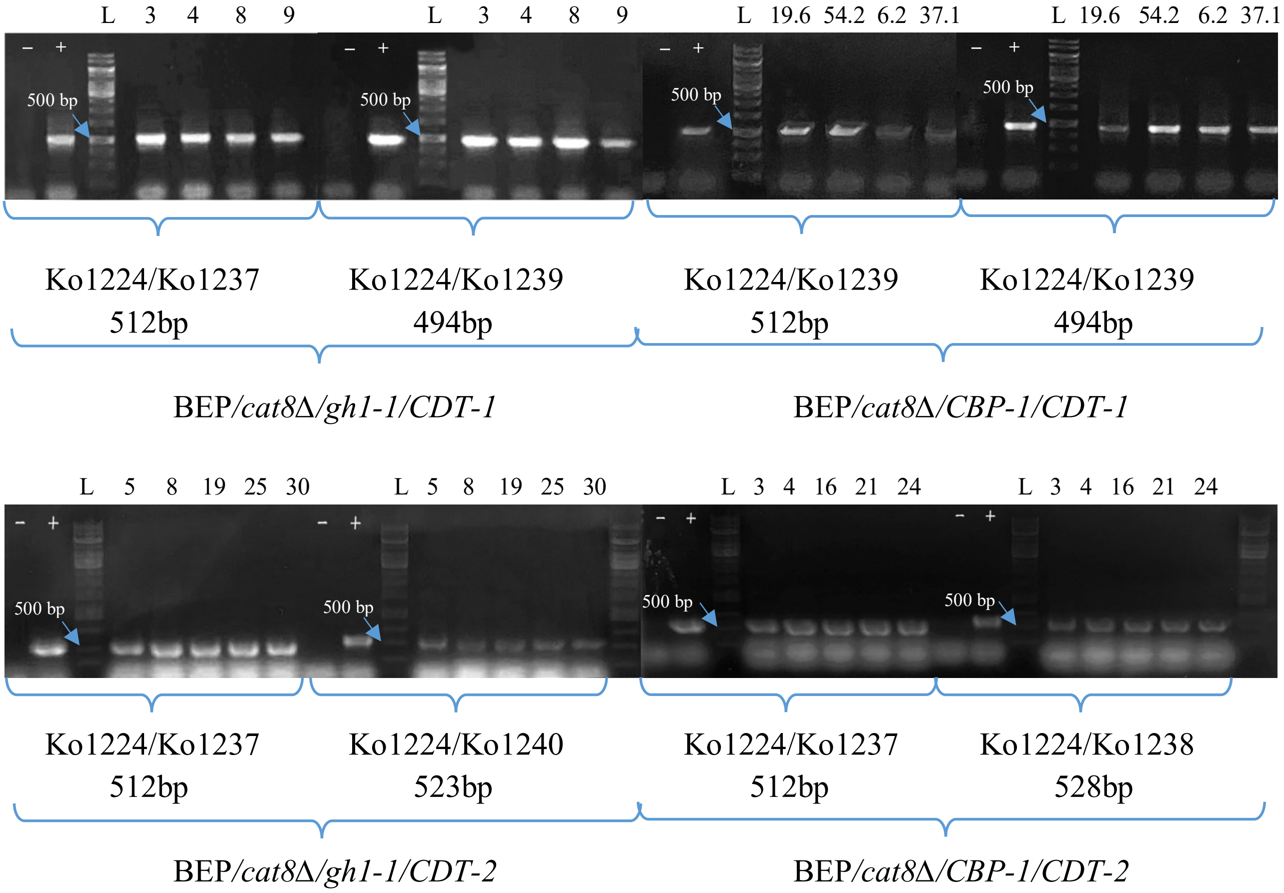

Fig. S2 Linear schemes of plasmids pUC19_pGAP_gh1-1_tGAP_NTC (A), pUC19_pGAP_CBP-1_tGAP_NTC (B), pUC19_pGAP_CDT-1m_tGAP_NTC (C), and pUC19_pGAP_CDT-2m_tGAP_NTC (D).

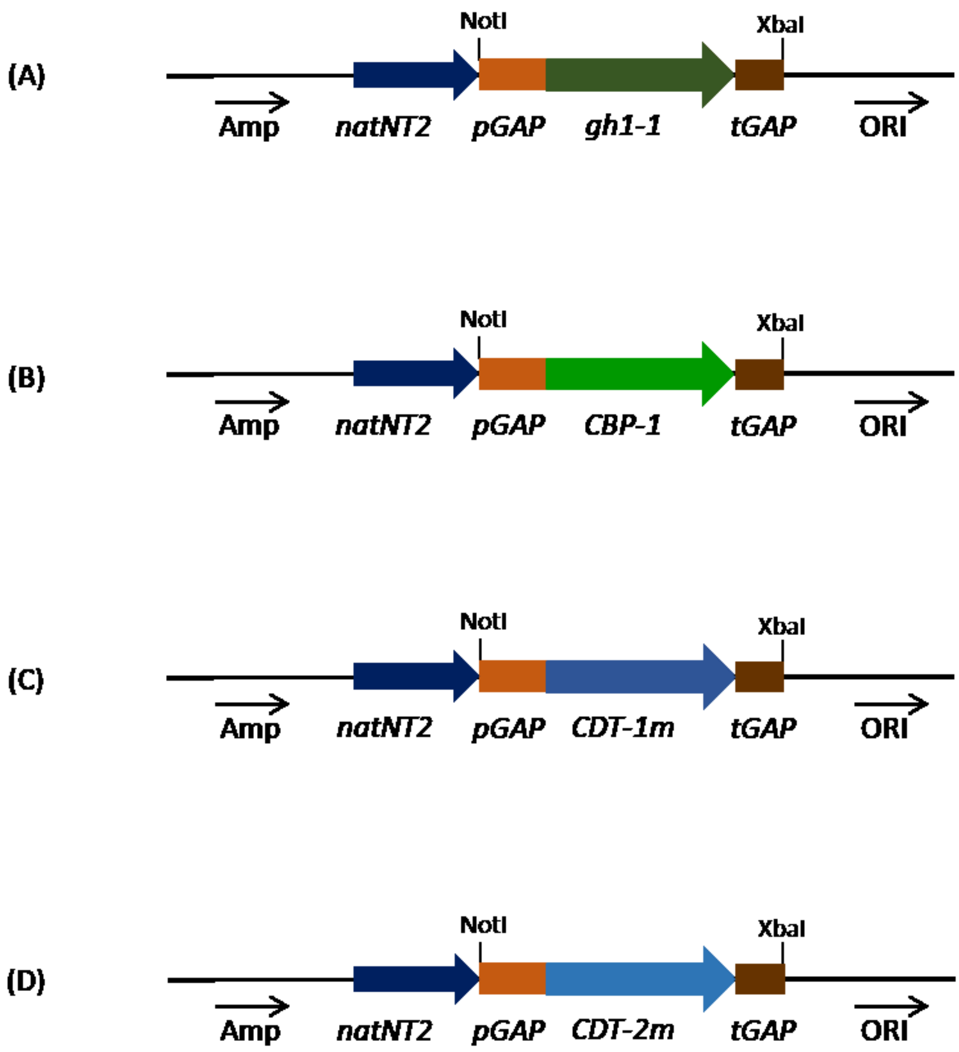

Fig. S3. Biomass accumulation *(A)* ethanol production *(B)* and sugar consumption *(C)* of *O. polymorpha* BEP/cat8Δ and recombinant strains with *gh1-1, CBP, CDT-1m, CDT-2m* gene during growth test on 2% cellobiose at 37°C *(A)* and alcoholic fermentation *(B, C)*10% cellobiose at 45°C. Data are shown as mean of two independent experiments.

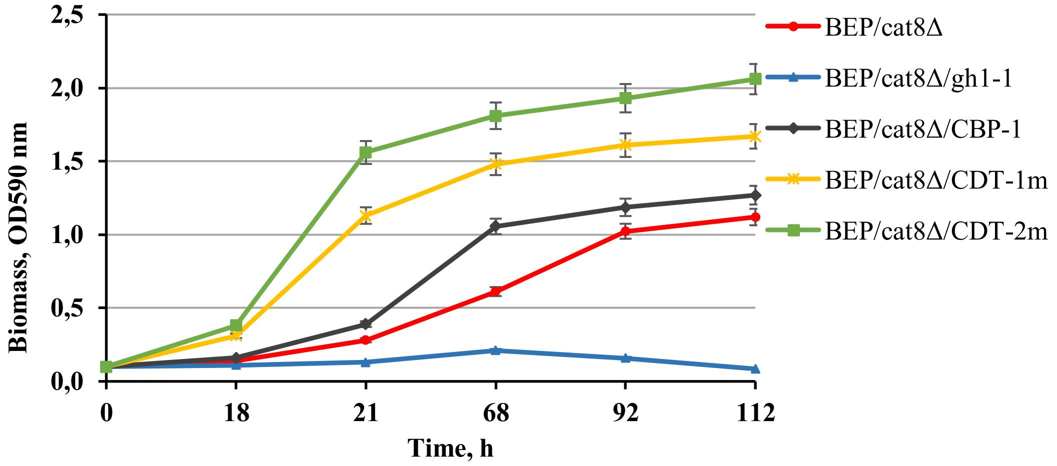

A

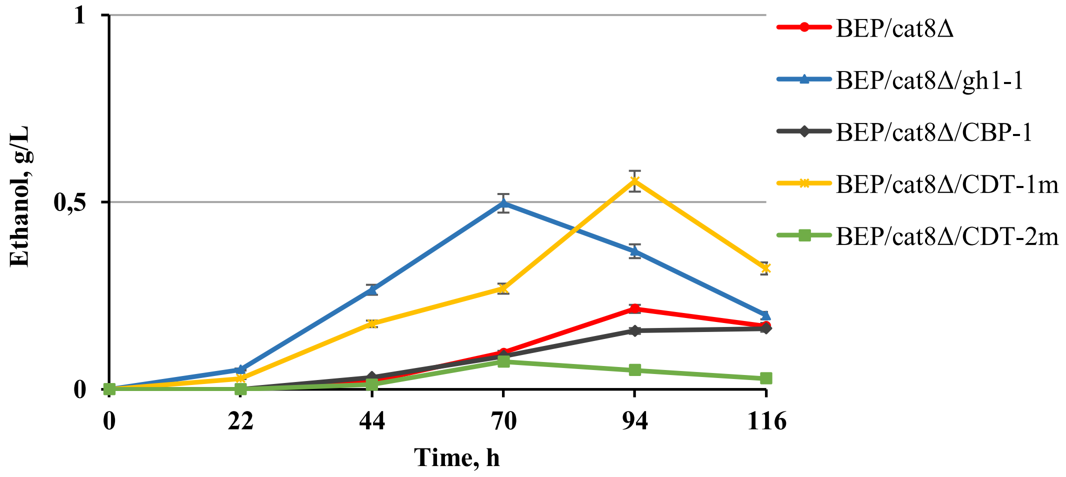

B

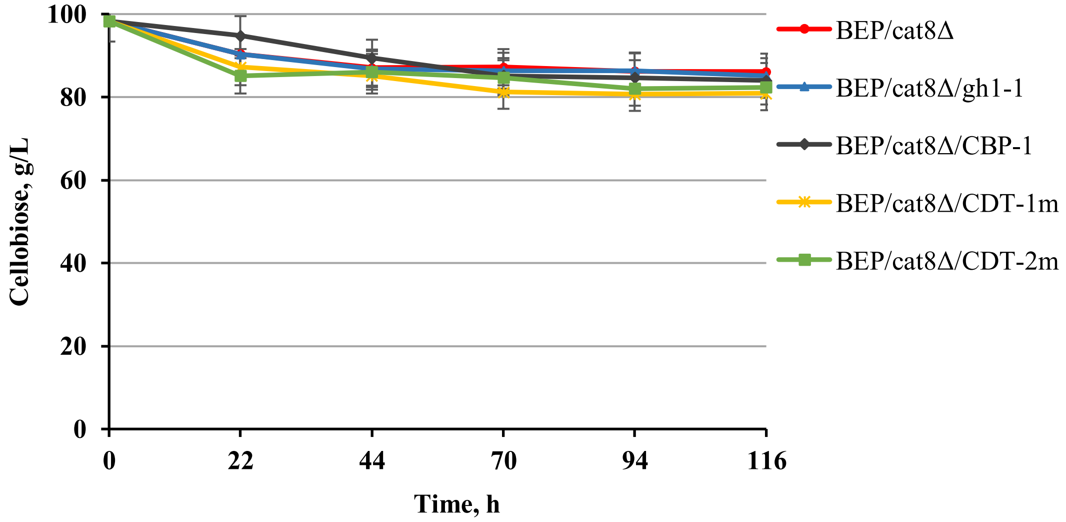

C
